# Adherence to behaviours associated with the test, trace, and isolate system: an analysis using the theoretical domains framework

**DOI:** 10.1186/s12889-022-12815-8

**Published:** 2022-03-22

**Authors:** Rachael J. Thorneloe, Elaine N. Clarke, Madelynne A. Arden

**Affiliations:** Present Address: Centre for Behavioural Science and Applied Psychology, Sheffield Hallam University, Sheffield, England

**Keywords:** COVID-19, Adherence, Test, trace, and isolate, Theoretical domains framework, Behavioural science

## Abstract

**Background:**

The UK’s test, trace, and isolate system are key measures to reduce the impact and spread of COVID-19. However, engagement with and adherence to guidance on testing, self-isolation, and providing details of contacts can be low and interventions are needed. This qualitative study aimed to identify the key factors affecting adherence to test, trace, and isolate behaviours using the Theoretical Domains Framework (TDF).

**Methods:**

We conducted six online focus groups between October 2020 and February 2021 with people living in Sheffield who came into close contact with others in work or social settings (*N* = 30). The focus groups explored capability, opportunity, and motivational barriers to adherence to test, trace, and isolate behaviours. Framework analysis was used to code the data into TDF domains.

**Results:**

There is a complex relationship between the factors affecting COVID-19 symptom identification, testing, and self-isolation. People who perceived significant barriers to testing and self-isolation were less likely to interpret potential symptoms as COVID-19, and perceiving barriers to self-isolation reduced the likelihood of requesting a test. Concerns about the negative consequences of self-isolation for themselves and others were common and also influenced willingness to pass on details of contacts. There was a lack of trust in the Test and Trace system, with people wanting further evidence of being at risk of infection.

**Conclusions:**

Communications and interventions to increase adherence to test, trace, and isolate strategies need to consider the interplay of these behaviours and their influences and target them collectively. Efforts to promote testing should focus on the range of barriers to self-isolation, especially increasing financial and practical support, and include new messaging to promote symptom identification.

**Supplementary Information:**

The online version contains supplementary material available at 10.1186/s12889-022-12815-8.

## Background

The COVID-19 pandemic has required that governments around the world develop systems for surveillance, contact tracing and case investigation [1]. While the details of these vary from country to country, they share some common features: (i) testing to determine where there are cases of positive COVID-19 infection; (ii) contact tracing to identify others who may have been exposed and are at high risk of infection; (iii) quarantine or self-isolation of known cases of COVID-19 infection, and of those who have been identified to have been at high risk of infection.

In the UK [2–5], the specific systems and guidance has changed over the course of the pandemic but in brief, people should self-isolate immediately, if they have any symptoms of COVID-19 (i.e., a high temperature, a new continuous cough or a loss or change to their sense of taste or smell), they should obtain a PCR (polymerase chain reaction) test to confirm whether they have COVID-19, and if the test result is positive, they should continue to self-isolate for a minimum period of 10 days as well as pass on details of close contacts to the national Test and Trace service. People should also self-isolate if identified as being at risk of infection and instructed to so by Test and Trace.

The success of the test, trace and isolate system relies on how well people: (1) identify COVID-19 symptoms; (2) request a COVID-19 test, (3) self-isolate if symptomatic, test positive for COVID-19, or if instructed to do so by Test and Trace, and (4) pass on details of close contacts to the Test and Trace service. However, adherence to these behaviours is low. The synthesised findings of 37 nationally representative surveys conducted with 53,880 people from March 2020 to January 2021 indicated that only 51.5% of participants correctly identified the main symptoms of COVID-19 [6]. Of those who reported experiencing COVID-19 symptoms, only 18% of people requested a test for COVID-19 within seven days and just 42.5% completely adhered to full self-isolation. Of those who had not experienced COVID-19 symptoms, 79.1% intended to share details of close contacts with the Test and Trace service if they tested positive or were asked to do so by the Test and Trace service.

These low levels of engagement/adherence are worrying because the test-trace-isolate system will remain a key strategy to reduce community infections. Even the most optimistic mathematical models of vaccine efficacy predict that vaccination alone will not be enough to stop the spread of COVID-19 and that non-pharmaceutical interventions will continue to be needed after vaccination programmes are complete [7].

To address the low levels of engagement and adherence to test, trace and isolate behaviours, we need to understand the key barriers and facilitators. This understanding will allow us to put the right services, support and interventions in place to increase adherence and therefore the success of the test-trace-isolate system. People need to have the capability, the opportunity, and the motivation (COM-B model of behaviour [8, 9]) to engage and adhere to test, trace, and isolate guidance. For example, in the context of self-isolation, the COM-B theory would predict that people need sufficient knowledge about exactly what they need to do and why, and they need to have the ability to plan and remember the correct action in the required situation (capability). They also need to believe in the value of self-isolation for themselves and others and feel confident that they can self-isolate and cope with any negative consequences (motivation); and have sufficient encouragement from others and support, including financial, practical and social support, to manage during a period of self-isolation (opportunity). The British Psychological Society have used the COM-B theory to inform their guidance for encouraging self-isolation [10].

Research has supported the importance of many of these factors, for example, a survey indicated that non-adherence to self-isolation was associated with lower perceived efficacy of lockdown measures, low perception of others’ adherence to lockdown rules and lower perceived severity of COVID-19, while higher adherence was associated with receiving help [11]. Similarly, a review of factors associated with adherence to quarantine during infectious disease outbreaks identified that knowledge of the disease and quarantine procedures (capability), perceived risk of the disease and benefits of quarantine (motivation), and social norms and practical issues (opportunity) as important [12].

However, there has been little research exploring adherence to test, trace and isolate behaviours using a qualitative approach. While large surveys are useful to indicate the prevalence of barriers and their relationships to intentions and behaviour, they cannot explore in detail why these barriers are relevant and important and how they might inter-relate with other aspects of people’s responses to living during the pandemic. This detailed understanding is important to inform interventions and to understand how different aspects of services and interventions might inter-relate.

The aim of this qualitative study was to explore individuals’ responses to the pandemic and how people were engaging with and adhering to the test, trace and isolate system.

## Methods

### Sampling and recruitment

Individuals living in Sheffield who reported that they were coming into close contact with others in social and work settings during different stages of the pandemic were recruited via a market research company (DJS). A purposive sampling strategy informed by the requirements of the funders was used to obtain a diverse demographic group (Table 1). Each focus group wave used a different sampling framework, to respond to emerging COVID-19 events, policy changes and priorities set by the funders.

**Table 1:** Sampling framework for the focus groups

Focus group wave and timepoint	Sampling framework
Focus group 1 and 2 (wave one) *October 2020*	• People aged 18 – 45 yearsPeople aged 18 – 45 years • Reported that they had been socialising with family and friends, inside and outside their home (e.g., using the ‘Eat out to help out scheme’), before the ‘rule of 6’ or ‘tier system’ restrictions came into effect
Focus group 3 and 4 (wave two) *December 2020*	• People aged 18 – 54 years • Reported that they ‘somewhat support’ or ‘somewhat oppose’ the new lockdown measures, with this item acting as a proxy for potential adherence difficulties during the second national lockdown
Focus group 5 and 6 (wave three) *February 2021*	• People aged 18 – 54 years • Reported being in an occupation that involved working in close proximity with others, but not in an occupation that regularly exposes them to diseases (e.g., health or social care)

### Data collection

**Fig. 1:**
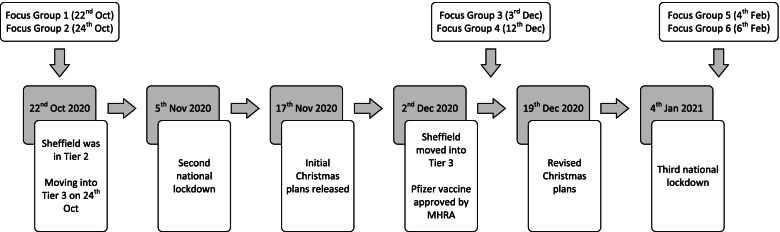
Focus group timeline

Six focus groups were conducted using an online platform, each lasting two hours. They were held in the evenings and at weekends to facilitate attendance. Focus groups were conducted across three time points during October 2020 and February 2021 (see Fig. 1), in order to respond to emerging COVID-19 events and/or policy changes. A semi-structured topic guide explored individuals’ capability, opportunity and motivation to undertake key COVID-19 preventative behaviours, including adherence to test, trace, and isolate behaviours (supplementary materials). All focus groups were conducted by two members of the research team (MA & RT), both of whom lived in Sheffield. Data were audio-recorded, transcribed, and anonymised. Informed consent was obtained from all participants prior to data collection. Ethical approval was received from Sheffield Hallam University (ER27692894).

### Data analysis

The four key behaviours relating to adherence to the test, trace, and isolate system were: (1) identifying COVID-19 symptoms; (2) requesting a COVID-19 test, (3) self-isolating if symptomatic, testing positive for COVID-19, or if instructed to do so by Test and Trace, and (4) passing on details of close contacts to Test and Trace. Framework analysis was used to code the data [13].

Two members of the team (EC & RT) read the transcripts to become familiar with the content, identify preliminary themes and key issues, and to identify the key behaviours (behaviours 1 – 4). Barriers and facilitators associated with the four key behaviours were initially mapped onto the relevant COM-B components during a rapid phase of analysis in line with the requirements of the work to inform local policy and communications. Later we then mapped directly to the relevant 14 TDF domains [14]. Text relating strongly to more than one TDF domain was coded in both. The types of statements under each TDF domain were analysed using inductive content analysis. Themes arising from the data (under each TDF domain) were identified to create sub-categories. All themes arising from the data were frequently compared between and within cases, and across different behaviours, to identify similar or contrasting themes, to identify disparities, and to explore patterns and connections between themes. During these later stages, links between perceived barriers and expected outcomes of one behaviour were found to influence the performance of different behaviours. One member of the team (EC) undertook the analysis, with the research team comprising of researchers with expertise in the COM-B model and TDF engaging in ongoing discussion to ensure appropriate interpretation, transferability, and credibility of the findings. Any disagreements in coding were resolved by revisiting the original text. Data were managed in NVivo.

## Results

### Sample characteristics

In total, 30 individuals living in Sheffield who reported coming into close contact with others in a work or social setting during different stages of the pandemic took part in the focus groups. Characteristics of the focus groups are shown in Table 2.

**Table 2:** Participant demographics per focus group (*N* = 30)

	Wave 1 (22-24/10/2020)		Wave 2 (03-12/12/2020)		Wave 3 (04-06/02/2021)		
***Focus group***	1	2	3	4	5	6	**Total**
***n***	4	5	3	8	6	4	30
Age (range)	20 - 44	18 - 45	37 - 51	20 - 54	21 - 54	32 - 40	18 - 54
Gender (*n*)							
Female	3	3	-	5	3	3	17
Male	1	2	3	3	3	1	13
Ethnicity (*n*)							
White	4	5	2	6	5	4	26
BAME	-	-	1	2	1	-	4
Employment status (*n*)							
Employed	2	3	3	5	6	4	23
Student	2	2^a^	-	2	-	-	6
Unemployed	-	-	-	1	-	-	1

^a ^Includes one part-time student who was also employed part-time

### Barriers and facilitators associated with COVID-19 symptom identification, testing, and self-isolation

**Fig. 2:**
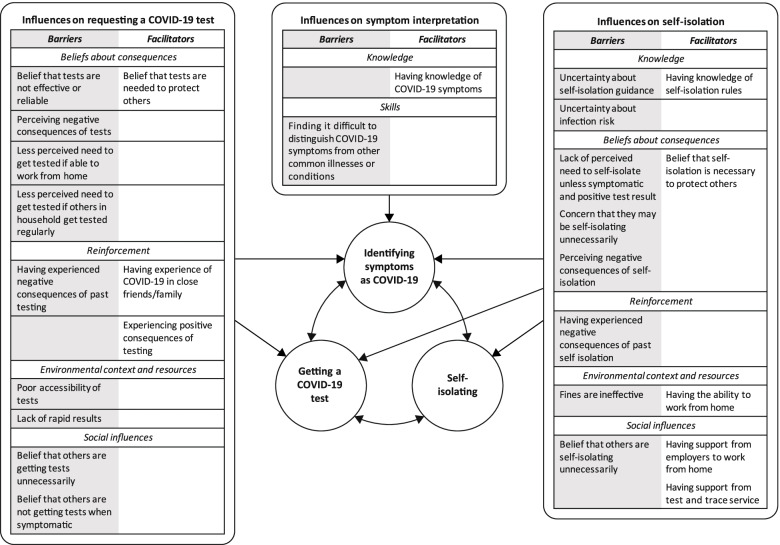
The influencing factors and relationships affecting COVID-19 symptom identification, testing, and self-isolation

The barriers and facilitators for identifying COVID-19 symptoms, requesting a COVID-19 test, and self-isolating are presented in Fig. 2. For each behaviour, results are presented according to each TDF domain, with explanatory themes provided alongside each theoretical domain. The factors associated with these three behaviours were highly linked, with the expected outcomes and ease of one behaviour (e.g., getting a COVID-19 test) influencing the performance of a different behaviour (e.g., self-isolating). This complex interplay is illustrated in Fig. 2.

#### Influences on the interpretation of COVID-19 symptoms

People generally knew about the key COVID-19 symptoms (high temperature, new continuous cough, a loss or change to sense of smell or taste) and this was an enabler to identifying symptoms as COVID-19. However, people found it difficult to distinguish COVID-19 symptoms from symptoms associated with other types of common illness (e.g., cold, flu) or conditions (e.g., ‘smoker’s cough’, allergies).

*“How do you know the difference between flu and Covid?” P18, FG4*.

Symptom ambiguity and confusion were barriers to seeking a test and self-isolating if symptomatic. Some believed this was resulting in an increase in case numbers.

*“…People are brushing things off saying oh it is just my normal cold that I’d get this time of the year and I don’t think people are getting tested and I think that’s why it is spreading a lot more at the minute.” P28, FG6*.

#### Influences on requesting a COVID-19 test

Perceptions of risk for oneself initially declined following the lifting of restrictions over the summer (post-first lockdown), even for those in high-risk groups, but increased again during the third lockdown. Changes in risk perception were related to their own and others’ experiences of COVID-19, as well as their own and others’ experiences of the lifting of the restrictions. Indeed, the perceived need to get a COVID-19 test straightaway *if symptomatic* to protect other people increased over the course of the pandemic. In the last wave of focus groups, more people knew someone who had got COVID-19 and had died from the virus, or knew people who had tested positive with COVID-19 after experiencing ambiguous cold-like symptoms. Some people experienced feelings of reassurance after requesting a test and receiving a negative result that they were not passing on the virus to their loved ones and the wider community.

*“I probably would now that I’ve heard that the people who have tested positive did have cold symptoms and not the cough and not the fever or the loss of taste or smell so yes”. P25, FG5*.

*“I did another test and everything frightens you so as long as – if I keep feeling these ways I will keep testing myself, yes” P23, FG5*.

However, the perceived need to get a test depended upon symptom interpretation, including symptom severity and longevity. Some people believed it was only necessary to get a test if they had more than one symptom of sufficient severity.

*“I’d say at least two out of the three to get a test, because you can wake up any day and have a cough, you can wake up another day and just be hot, so I think if you show at least two out of the three then that’s the only time where I’d go and get one.“ P9, FG2*.

Some people also believed that symptoms needed to persist for a sufficient length of time, with some people self-isolating at home and waiting to see if symptoms improved over time before requesting a test.

*“…I wouldn’t [get a test] straight away to be fair, no, I wouldn’t straight away. I would obviously stay at home and if it went on for a couple of days then I would get, you know, I would get tested”. P16, FG4*.

The perceived need to get tested was also influenced by the presence of others in their household getting tested. Some people discussed how members of their household getting regular lateral flow tests (e.g., testing in schools and colleges) made them feel safer. They believed they would know if there was COVID-19 in their household, without having to rely on detecting ambiguous symptoms.

*“…I felt safe knowing that she gets tested a lot and she’s coming home to an environment where we’re okay…” P27, FG6*.

However, for some, even experiencing distinct symptoms was insufficient to request a test.

*“…If I had a temperature and a persistent dry cough then it would prompt me to* ***maybe go*** *and get a test.” P2, FG1*.

If distinct symptoms were experienced, some reported that they would self-isolate without seeking further confirmation from a test as they were able to work from home.

*“I personally wouldn’t go and get one but that’s just because I can work from home, so rather than wasting one I think I’d just self-isolate for fourteen days…other people need it so save it for someone else, I can still work.“ P8, FG2*.

This was partly due to a lack of trust in the effectiveness and reliability of tests in detecting COVID-19. They considered their own interpretation of symptoms as sufficient proof of having COVID-19.

*“…If I had like the direct symptoms, like cough, fever, maybe not so much the smell and taste but yes, if I had direct symptoms and pretty much I could say it was Covid then I would self-isolate, I don’t think you need a test for that.“ P1, FG1*.

*“…The tests have very high false positives and they’ve even got false negatives as well. So you can’t, you wouldn’t be able to rely on the test anyway…” P18, FG4*.

Poor accessibility of COVID-19 tests and lack of access to rapid test results were also barriers to requesting a COVID-19 test.

*“Like as much as I’d want to do my part in it, is it worth going to all the trouble because it’s long queues to get a test. You might have to go to [a different town] for a test, like, it’s not a local kind of thing most of the time, sometimes.” P1, FG1*.

For those who were unable to work from home, they wanted to be able to get a test and receive the results quickly, to end self-isolation and return to work.

*“I think the testing needs to be either getting hold of it quicker and you get your results back quicker so you can get back to work quicker, but I’ve not known anyone can get a test.“ P6, FG2*.

*“Yes, yes definitely [I would want to get tested quickly], just so that I can rule it out and get back to work as soon as possible…” P19, FG4*.

However, people would be unlikely to request a test if experiencing ambiguous symptoms, due to the negative consequences of self-isolation on their ability to work and their income.

*“Like, if I just felt like I had a common cold, as bad as it sounds, I don’t think I’d go to the effort of like not working and possibly, like, losing money, getting a test” P7, FG2*.

#### Influences on self-isolation

Information about infection risk from Test and Trace or the NHS COVID-19 contact tracing app encouraged people to re-interpret previously ambiguous symptoms.

*“…She got a notification saying, like, where you’ve been, you’ve been in contact with someone…that is how she knew she had it because before she was just like… you know, it’s just a cold.” P1, FG1*.

However, although information about infection risk was necessary, it was insufficient by itself to encourage self-isolation. Self-isolation was viewed as necessary to protect others, however; people wanted more proof that they had been in close contact with someone with COVID-19. Some discussed how they would only self-isolate after being instructed to do so by Test and Trace if they also experienced distinct symptoms or if they had a positive COVID-19 test result.

*“…If I then felt ill I would go and get a test and if it came back positive obviously I would stay in but if I got a phone call saying oh you’ve been sat with someone who’s got Corona, unless I am feeling ill, I am not going to do it.“ P4, FG1*.

*“I believe that if I received a phone-call saying that I’ve been in contact… I’ll go and get a test. I’ll go and get a test. If it comes back positive, I’ll self-isolate. If it comes back negative, I won’t. Just keeping it simple.“ P10, FG3*.

This was due to the negative consequences self-isolation had on their ability to work, their income, and their mental health and well-being.

*“I would only self-isolate if I’d had the test because I feel you’re staying in for ten days and it potentially might be for nothing.“ P2, FG1*.

*“…Am I going to give up two weeks of pay just because I’ve possibly been in the same room as someone? No but then if I start to feel ill then yes, I’d get a test but at first, no.“ P1, FG1*.

Similarly, some people chose not to download the NHS COVID-19 contact tracing app, due to the potential that they could be told to self-isolate without sufficient proof of infection risk. The functionality of the app (e.g., being unable to log out of venues) was viewed as increasing the risk of being told to self-isolate unnecessarily i.e., if the period of infection risk did not match the time they were actually present at the venue.

*“Well if you then have to self-isolate then your partner has to self-isolate and then suddenly, like, you’re affecting their workloads, stresses, everything else and you think to yourself as long as you’re being safe and you’re sticking to the rules, I don’t need this Test and Trace app and that was my personal reason why I never downloaded it.” P27, FG6*.

*“…Why would I want to go from A to B, very briefly, and then someone, a few hours [later], enters my B zone, and I get a phone-call. Who wants that?” P10, FG3*.

Due to the negative consequences of self-isolation on themselves and others, people did not think it was necessary to self-isolate for a minimum of 10 days if they were not experiencing symptoms or had a negative test result. People wanted a shorter self-isolation period.

*“Fourteen days is quite a lot…it damages his education and I think I’d rather give the parents the choice as whether to isolate or not, you know after a week if none of them have got symptoms could they go back or something like that.“ P17, FG4*.

*“I think now they should be, like, saying to people, look, you know, give it a day or two, you know, take what you’d take if you had a flu, you know, and then see if you do get better…maybe after three or four days, if you’re still feeling absolutely shocking, go get a test.” P11, FG3*.

Whereas self-isolation fines were viewed as an ineffective strategy to promote self-isolation, the ability to work from home and having support from employers and from the Test and Trace service to self-isolate were facilitators.

*“Yes, they [employers] have been very supportive… and we do get paid for being off.” P6, FG2*.

#### Influences on providing details of close contacts to Test and Trace

The barriers and facilitators for passing on details of close contacts to Test and Trace are presented in Table 3. For each behaviour, results are presented according to each TDF domain, with explanatory themes and exemplar quotes provided alongside each theoretical domain. There were some similarities in the factors associated with passing on details of close contacts and the factors associated with symptom interpretation, getting a COVID-19 test, and self-isolation. People were aware of the Test and Trace guidance for passing on details of close contacts to contact tracers (*knowledge*), however, some discussed the difficulty in identifying ‘close contacts’, especially for those who were regularly coming into close contact with other people (*skills*). People believed contact tracing was an important strategy to protect other people but there were concerns about the potential negative impact self-isolation could have on their contacts’ ability to work and their income, especially for those who were self-employed (*beliefs about consequences*).

**Table 3:** Barriers and facilitators for passing on details of close contacts to Test and Trace

TDF domain	Theme	Exemplar quotes
Knowledge	Having knowledge about the need to pass on details of close contacts (F)	“If I were to test positive now I could say I went out and met my friend for a walk last week and I’d have to notify them” P28, FG6
Skills	Not having the ability to identify close contacts (B)	“…It’d be quite difficult to list who I’ve been in contact with…when I’m in [work] there’s like forty, fifty people” P8, FG2
Beliefs about consequences	Belief that passing on details of close contacts is important to protect others (F)	“I think if you know you’ve been in contact with somebody it’s only right to let, you know, let someone know that you’ve been in contact with that person. We don’t know to what lengths Covid can have impacts on [us do we]…” P2, FG1
	Having concerns about data privacy (B)	“I wouldn’t give anyone’s information to anybody, not without their consent first anyway because it’s what they call it, the privacy and stuff like that.“ P18, FG4
	Perceiving negative consequences of passing on details of close contacts (B)	“Most of the people that I know are self-employed and the businesses are struggling anyway since we’ve been allowed to reopen and if they have to stop working again and still pay staff wages instead of their own hours, I don’t think their businesses would survive, so I would feel really bad about giving their details over.“ P7, FG2
		“I’d always ask just to make sure, if they wanted me to then I would but then if not, then I’m not going to because then they’re off work and there’s no income and I’d feel awful because they’re not earning what they should earn.“ P5, FG2
	Concerns about the efficiency of Test and Trace contacting people (B)	“So it’s not that I wouldn’t necessarily engage with track and trace but I don’t actually think that they end up getting, passing that information on in like a timely manner anyway, so I think I’d probably just give the people I know I’d been in contact with a call so they could, could isolate.” P16, FG4
Social role and identify	Feeling a moral duty to pass on details of close contacts (F)	“It’s almost like a duty of care isn’t it?” P2, FG1
Emotion	Feelings of anticipated regret about the perceived negative consequences of passing on details of close contacts (B)	“I’d feel awful because they’re not earning what they should earn. I just feel like it’s not fair to them people that have been in contact for like, two minutes, like, you’ve just walked past them or you’ve been with them a couple of hours…” P5, FG2

*B* barrier, *F* facilitator

There were also some differences in the factors associated with passing on details of contacts to Test and Trace. Some people discussed how they would inform their close contacts themselves, rather than pass on their details to Test and Trace, and this was due to concerns about data privacy and concerns that contact tracers do not always get in contact with people in an efficient and timely manner (*beliefs about consequences*).

## Discussion

We undertook an in-depth qualitative study exploring individuals’ responses to the pandemic and how people were engaging with and adhering to the test, trace, and isolate system. We found that there is a complex relationship present between engagement/adherence with key behaviours related to test, trace and isolate. An important finding is that the expected outcomes and ease of those outcomes for one behaviour influenced the performance of another behaviour. Those who perceive barriers and negative outcomes for getting a COVID-19 test are less likely to interpret symptoms as COVID-19. Similarly, those who perceive barriers and negative outcomes for self-isolation are less likely to perceive symptoms as COVID-19 or get a test. Previous research has investigated test, trace, and isolate behaviours as separate behaviours [10] but this approach misses the complex relationships present between these linked behaviours and their influences.

The COM-B model is a useful framework for understanding the full range of factors within the system that might influence a behaviour [8, 9], including adherence to COVID-19 protective behaviours [10, 11]. The current study demonstrates the importance of qualitative research in exploring the complex adherence challenges present for related behaviours, such as those involved in test, trace, and isolate systems.

Interventions to increase levels of COVID-19 testing and self-isolation need to consider the interplay of these behaviours and their influences. Although the Behaviour Change Wheel (BCW) [8, 9] emphasises the importance of identifying and operationalising the key target behaviour(s) (i.e., when, where, what, whom), the current findings suggest that it is also important to consider *how* different behaviours are related. For example, to create an effective intervention that promotes COVID-19 testing, we must understand what people need to do before requesting a COVID-19 test (i.e., identify symptoms as COVID-19) and what people need to do after getting a COVID-19 test (i.e., self-isolate). After identifying the related behaviours and the sequence in which they need to occur, it is then important to examine the range of *factors* that influence the behaviours. For example, as we know that lack of available COVID-19 tests is a barrier to getting tested, unless interventions address this barrier then people will remain unlikely to get a test, and importantly, be unlikely to do related behaviours that precede it (i.e., identify symptoms as COVID-19) or follow it (i.e., self-isolate). There has been a call for more research applying a complex adaptive systems approach to understand the complexity of behaviour change [15] and this research supports the role of qualitative research in understanding this complexity.

Symptom appraisal is complex and influenced by a range of factors [16], which this study supports. People had a good understanding of the key COVID-19 symptoms, but the ambiguous nature of these symptoms made it less likely that symptoms were perceived and interpreted as COVID-19. According to Leventhal’s Common Sense Self Regulation model [17], people have their own lay prototype or model of illness, based on their own experiences. One common prototype will be based on the common cold or flu, with individual's past experiences building a prototype of expected symptoms, outcomes, and required behaviours. The symptoms associated with COVID-19 share many similarities with symptoms associated with colds/flu, and so according to the model, the experience of ambiguous COVID-19 symptoms may activate the cold/flu prototype, resulting in individuals being less likely to believe they have COVID-19 even when experiencing symptoms and thus less likely to seek a test and/or self-isolate. Previous research has demonstrated the importance of lay representations of illness influencing behavioural responses to different health threats [18]. Our findings suggest that people’s perceptions about the expected outcomes and ease of seeking a test and/or self-isolating may reinforce people to think of their symptoms as an acute illness. Our findings are consistent with those of a recent study on COVID-19 symptom recognition, which found that people were most likely to attribute symptoms to COVID-19 when symptoms were severe and had lasted for some time, when more than one symptom was present, and when there was a perceived risk of exposure due to contact with others [19]. The situation is likely to be even more complex given reports of a different range of symptoms being associated with the delta variant [20], as well as the symptom profiles of other/future variants, such as omicron.

Importantly, our findings show that the expected outcomes and ease of getting a COVID-19 test or self-isolating influence symptom appraisal. Self-isolation could have a negative impact on people’s ability to work and their income, and this was a key barrier which influenced symptom appraisal. Previous research has demonstrated that socio-demographic factors, including socioeconomic deprivation, influence symptom appraisal and help-seeking behaviours [21]. There is low uptake of COVID-19 testing in areas of deprivation [22], and findings of the current study suggest this might be due to self-isolation increasing the potential for additional employment/financial burdens. Note that this research was conducted before the widespread deployment of lateral flow tests, and it seems likely that different types of tests will add to the confusion about symptoms and PCR testing [23].

### Strengths and limitations

All participants were Sheffield residents and thus the findings may not be transferable to other areas of the UK. However, research has demonstrated Sheffield to be a microcosm of the UK in terms of sociodemographic inequalities, including economic, social, and health [24]. A limitation is that engagement and adherence were self-reported rather than objectively measured. However, our qualitative study provides new insights into the factors influencing adherence to test, trace, and isolate behaviours which have been missed in previous survey studies. Our findings will be valuable for informing future quantitative studies, which could explore the extent to which these relationships are prevalent across the UK and whether they differ for different population groups. To our knowledge, there is a lack of research on the impact of the COVID-19 vaccine rollout on adherence to test, trace, and isolate behaviours. This should be explored in future research, as should the impact of the widespread use and recommendation of different types of tests (lateral flow and PCR).

### Implications for interventions

Increasing engagement and adherence to the test, trace, and isolate system requires interventions that address symptom interpretation, testing, and self-isolation collectively, rather than separately. Efforts to increase testing need to address the barriers for requesting a test, but also need to simultaneously promote symptom interpretation and address barriers to self-isolation. This may include individual-level interventions that support people to make a plan for what they need to do if they notice symptoms or are asked to self-isolate, in advance of need. Communications and messages should emphasise that the best action if unsure about symptoms is to get tested, and ensure that communications emphasise how to get tested quickly and easily. Improved policies need to be developed and publicised to support people financially and practically to seek a test as soon as possible, even with mild or ambiguous symptoms, and to self-isolate. Increasing and widely publicising policies and practices within organisations that encourage testing and self-isolation, alongside messages that emphasise data privacy policies, may also increase willingness to pass on details of contacts to Test and Trace.

## Conclusions

This study has identified a complex relationship between the factors affecting COVID-19 symptom identification, testing, and self-isolation. The expected outcomes and ease of those outcomes for one behaviour (i.e., getting a test or self-isolating) influenced the performance of another behaviour (i.e., symptom interpretation). To our knowledge, this research study is the first to examine the interplay of factors influencing adherence to test, trace, and isolate behaviours. Our qualitative methods have produced a richer understanding of the relationships present between symptom identification, testing and self-isolation, and suggests that interventions are more likely to be successful if they address these behaviours and their influences collectively, rather than separately. These findings can be used to develop multi-faceted interventions and communications that work together to address these behaviours simultaneously.

## Supplementary Information

**Additional file 1. **Topic Guide.

## Data Availability

The data and materials used in this study are available from the corresponding author on reasonable request.

## Abbreviations

COM-B: Capability, Opportunity and Motivation model of Behaviour
PCR: Polymerase Chain Reaction
TDF: Theoretical Domains Framework
