# Supplementary material for: Adherence to behaviours associated with the test, trace, and isolate system: an analysis using the theoretical domains framework
Source: BMC Public Health. 2022 Mar 22;22:567. doi: 10.1186/s12889-022-12815-8 (PMC8938733; doi:10.1186/s12889-022-12815-8)
Supplement: Supplementary file 1 — Additional file 1. Topic Guide. [file 12889_2022_12815_MOESM1_ESM.docx]

**Supplementary Materials**

**Topic Guide**

***This paper represents the analysis of only part of the data***

Introductions

- Tell us a little about yourself (job, where you live and who with)?
- What do you understand about COVID/coronavirus?
- Have you or anyone close to you had COVID?

Causes of coronavirus

- What do you understand about the origins/causes of coronavirus?
- Where have you got this information?
- How much do you trust these sources? Are some more trusted than others?

Perceptions of personal risk

- How likely do you think it is that you might catch COVID?
- What are you doing that might increase your risk?
- What are you doing to try to reduce your risk?
- How severe do you think it would be if you caught COVID?
- Some people seem to be experiencing longer term impacts of COVID (long-covid). How likely do you think this is to affect you?

Risks of transmission

- How likely are you to pass COVID on to others (family, friends, others)?
- What are you doing to avoid this?
- Are you worried about passing it on? About whom are you worried?
- Are your friends and family taking steps to avoid passing it on to you?

Households/bubbles/rule of 6

- Who is in your household?
- Are you part of a bubble? Who is in that bubble with you?
- What are you allowed to do in your household/bubble that you should not do with people outside?
- Why is there a focus on households/bubbles? How important is this?
- What is the rule of six?
  - What do you think about it?
  - How important is it to follow?
  - How fair is it?

Scenarios

Take a look at each of these scenarios [images of social distancing challenges in different settings, inside and outside, and images of other behaviours (e.g., sharing cigarettes, drinks, food) that may occur when socialising with family/friends)]

- Would you have any concerns in any of these settings?
- Are there any that you would avoid entirely?
- Would you want to take any additional precautions in these settings? What? Why?
- What are the most potentially risky situation you have found yourself in?
  - Why was it risky?
  - How did that make you feel?
  - Would you avoid being in that situation again?
- Have your friends or family been in these situations - what did they do?

Testing and self-isolation

- What are the main symptoms of COVID to look out for?
- What should you do if you or someone in your household has symptoms?
- How severe do these symptoms need to be for you to take action?
- Where would you seek information if you were unsure whether you or a member of your family were experiencing COVID symptoms?
- How/where would you get a test? Do you have any concerns about this?
- What are people supposed to do if they are self-isolating?
- How easy would it be for you to self-isolate for 14 days?
  - Have you made any plans about how you would do this/ who you could ask for help?
- Thinking about your close friends or family - do you think they would get tested/self-isolate? Why/why not?
- What would help people to be more willing to self-isolate?
- Would you be happy to talk to test and trace about your contacts?
- Would you be OK with your contacts identifying you to test and trace? Would your friends be happy?

NHS contact tracing app

- Have you downloaded the NHS contact tracing app or are you planning to?
  - Why/why not?
  - How important do you think it is?
  - Do you have any concerns about it? What?

COVID-19 vaccine

- What do you understand about the COVID-19 vaccine?
- Where have you got this information?
- Has this information impacted on how you are feeling, what you are doing or planning to do?
- How much do you trust these sources? Are some more trusted than others?
- Would you like to receive the COVID-19 vaccine?
  - Why/why not?
  - How important do you think it is?
  - Do you have any concerns about it? What?
  - Do you think others should receive the COVID-19 vaccine? Who?

Information and communication

- Who would you like to provide you with information/communications about COVID?
- How would you like to receive these?
- What would make you trust the messages?
- What would make you dismiss/not trust the messages?
- What do you think Sheffield City Council should do?

Preventative behaviours

- What are the ways that coronavirus infections are said to be prevented/reduced?
- Why/how do these reduce risks?
- How have you found out about these? How much do you trust these sources?
- Which of these are most/least important?
- Have messages about prevention been consistent?
- Is it easy or difficult to understand exactly what you should be doing and when?
  - What might help to explain this more clearly?
- Handwashing
  - What makes this easier to do?
  - What makes this more difficult to do?
  - Tell us about a time that you didn't wash your hands - why? What got in the way?
- Face coverings
  - What makes this easier to do?
  - What makes this more difficult to do?
  - Tell us about a time that you didn't wear a face covering when you should - why? What got in the way?
- Social distancing
  - What makes this easier to do?
  - What makes this more difficult to do?
  - Tell us about a time that you didn't social distance - why? What got in the way?

Lockdown

- How did you find the period of lockdown?
  - Was there anything that made it easier/more difficult?
- How likely is it that we'll have another lockdown?
- How would you feel about another lockdown?
- What needs to happen to prevent another lockdown?
- Have you made any preparations for another lockdown?
